# An improved whole life cycle culture protocol for the hydrozoan genetic model *Clytia hemisphaerica*

**DOI:** 10.1242/bio.051268

**Published:** 2020-11-05

**Authors:** Marion Lechable, Alexandre Jan, Axel Duchene, Julie Uveira, Brandon Weissbourd, Loann Gissat, Sophie Collet, Laurent Gilletta, Sandra Chevalier, Lucas Leclère, Sophie Peron, Carine Barreau, Régis Lasbleiz, Evelyn Houliston, Tsuyoshi Momose

**Affiliations:** 1 Sorbonne Université, CNRS, Institut de la Mer de Villefranche, UMR7009 Laboratoire de Biologie du Développement de Villefranche-sur-Mer (LBDV), 06230 Villefranche-sur-Mer, France; 2 Sorbonne Université, Institut de la mer de Villefranche, FR3761, Centre de Ressources Biologiques Marines (CRBM), Service Aquariologie, 06230 Villefranche-sur-Mer, France; 3 California Institute of Technology, Division of Biology and Biological Engineering, and the Howard Hughes Medical Institute, 1200 E California Blvd, Pasadena CA 91125, USA

**Keywords:** Animal culture, Cnidarian, Developmental biology, Genetics, Jellyfish

## Abstract

The jellyfish species *Clytia hemisphaerica* (Cnidaria, Hydrozoa) has emerged as a new experimental model animal in the last decade. Favorable characteristics include a fully transparent body suitable for microscopy, daily gamete production and a relatively short life cycle. Furthermore, whole genome sequence assembly and efficient gene editing techniques using CRISPR/Cas9 have opened new possibilities for genetic studies. The quasi-immortal vegetatively-growing polyp colony stage provides a practical means to maintain mutant strains. In the context of developing *Clytia* as a genetic model, we report here an improved whole life cycle culture method including an aquarium tank system designed for culture of the tiny jellyfish form. We have compared different feeding regimes using *Artemia* larvae as food and demonstrate that the stage-dependent feeding control is the key for rapid and reliable medusa and polyp rearing. Metamorphosis of the planula larvae into a polyp colony can be induced efficiently using a new synthetic peptide. The optimized procedures detailed here make it practical to generate genetically modified *Clytia* strains and to maintain their whole life cycle in the laboratory.

This article has an associated First Person interview with the two first authors of the paper.

## Abstract (precis)

**Summary:** We developed a reliable whole life cycle culture method for the hydrozoan jellyfish *Clytia hemisphaerica*, a model animal for genetics and developmental biology.

## INTRODUCTION

Choice of model species is a fundamental decision in biological research. Standard models such as *Drosophila*, mice, or *Caenorhabditis elegans* benefit from clear advantages of accumulated knowledge, established laboratory strains and experimental techniques. Non-standard model animals can also contribute, for instance, by providing insights into animal evolution, diversity and their interactions with the natural environment, as well as access to some biological processes absent or difficult to study in standard models (Cook et al., 2016; Goldstein and King, 2016). When using non-standard models, one significant hurdle is to establish standardized methods for raising and maintaining animals in the laboratory and controlling reproduction to obtain reliable embryonic and post-embryonic developmental stages. We describe here a robust laboratory culture method for the hydrozoan jellyfish *Clytia hemisphaerica* (Cnidaria), which has now become an accessible animal model to address a wide range of biological questions (Houliston et al., 2010; Leclère et al., 2016).

Cnidaria diverged early during animal evolution from the clade Bilateria, which includes almost all standard experimental model species. Adult bodies of most cnidarians are organized radially and comprise only two (diploblast) rather than three (triploblast) germ layers. They nevertheless possess nervous systems, muscles, and sensory organs that have evolved in parallel to those in found in Bilateria. Comparative studies between cnidarians and bilaterians are thus providing insights into how metazoans have acquired their current diversity. A variety of cnidarian species has been employed for experimental studies, in particular for developmental biology and evo-devo research. Notable examples are the anthozoan *Nematostella vectensis* (sea anemone) and the hydrozoan polyp species *Hydra* (freshwater) and *Hydractinia* (saltwater), (Galliot, 2012; Plickert et al., 2012). Laboratory culture of these three species is simplified by the absence of a pelagic medusa (jellyfish) stage, a life-cycle stage characteristic of many cnidarian species in Medusozoa (including Hydrozoa, Scyphozoa, Cubozoa and Staurozoa) but absent in its sister clade Anthozoa. Indeed, culturing the medusa stage in the laboratory is challenging. Scyphozoan jellyfish of the popular species *Aurelia aurita* (Gold et al., 2019) are easy to generate from polyps and to maintain in aquaria, but the life cycle is long and difficult to complete (Spangenberg, 1965). A full life cycle has also been obtained in captivity for the direct developing (i.e. polyp-free) scyphozoan jellyfish, *Pelagia noctiluca* (Lilley et al., 2014; Ramondenc et al., 2019) In contrast, hydrozoan medusae are generally smaller with shorter lifespans, and thus attractive as laboratory jellyfish models.

**Fig. 1.**
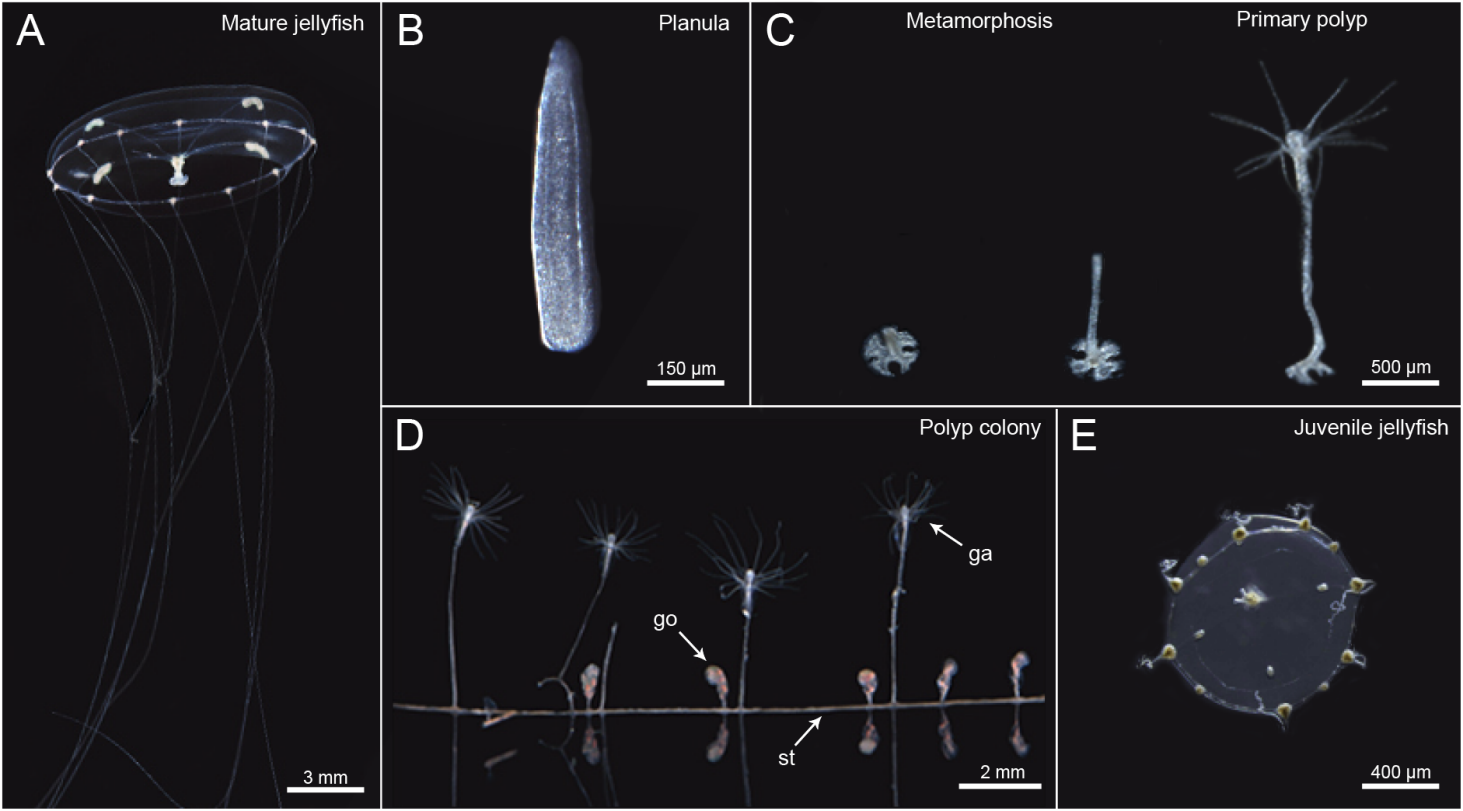
**Stages of the *Clytia hemisphaerica* life cycle**. The *Clytia* life cycle consists of polyp stage, jellyfish stage and embryo-planula larva stage. (A) Adult jellyfish survive several weeks in the laboratory and spawn eggs or sperm, upon light cues. (B) Fertilized eggs develop into planula larvae. (C) Planulae undergo metamorphosis when they recognize a solid surface covered by biofilm, which can be induced by GLW-amide neuropeptide added to sea water in the laboratory conditions. (D) A primary polyp extends a stolon (st) horizontally and make a colony with multiple feeding polyps (gastrozooids, ga) and medusa-budding polyps (gonozooid, go) with sufficient feeding, which are all genetically clonal. Gonozooid forms medusae buds, which are detached as juvenile jellyfish. Background particles were eliminated for representation purposes.

We have developed *Clytia hemisphaerica* (Linnaeous, 1767) as a jellyfish model, primarily motivated by its suitability for developmental biology and cell biology. *Clytia* is transparent throughout the whole life cycle (Fig. 1), an advantageous feature for microscopy. Daily release of gametes from jellyfish (Fig. 1A) can be controlled simply by the dark–light cycle. The life cycle can be completed in as little as 2 months in the laboratory. Fertilized eggs develop to form a mature planula larva in 3 days (Fig. 1B). Upon appropriate bacterial cues, conveniently substituted by adding synthetic GLWamide neuropeptides in sea water, they settle on a suitable substrate and metamorphose into primary polyps (Fig. 1C). From each primary polyp, a colony composed of polyps specialized for feeding (gastrozooids) and budding medusae (gonozooids) then develops vegetatively by extension of the connecting stolon network (Fig. 1D). Juvenile medusae (Fig. 1E) bud continuously from the gonozooids if the colony is well fed, and reach adult size (about 10 mm bell diameter) and sexual maturity in 2–3 weeks in aquarium conditions (Fig. 1A).

Available molecular and genetic resources include a whole genome sequence assembly and staged transcriptomes (Leclère et al., 2019; available on the ‘Marimba’ database http://marimba.obs-vlfr.fr). Efficient methods for gene function analysis have made *Clytia* an attractive genetic animal model, notably Morpholino antisense oligo and mRNA micro-injection into eggs prior to fertilization (Momose and Houliston, 2007), and more recently highly efficient CRISPR/Cas9-mediated gene KO (Momose and Concordet, 2016; Momose et al., 2018).

Here we detail the laboratory culture conditions and parameters affecting growth and development of *Clytia* over the whole life cycle. The culture system and tanks have been optimized for each stage. Daily feeding of *Artemia salina* nauplii is sufficient to maintain polyp colonies and medusae. We show how appropriate feeding regimes are critical factors for rapid growth in the laboratory, especially at very early medusa and primary polyp stages. The reliable culture system described here provides a solid basis for genetic studies in *Clytia,* for instance using mutants generated by CRISPR/Cas9 or emerging transgenesis technologies.

## RESULTS

### Overview and rationale of the aquarium system for *Clytia* culture

**Fig. 2.**
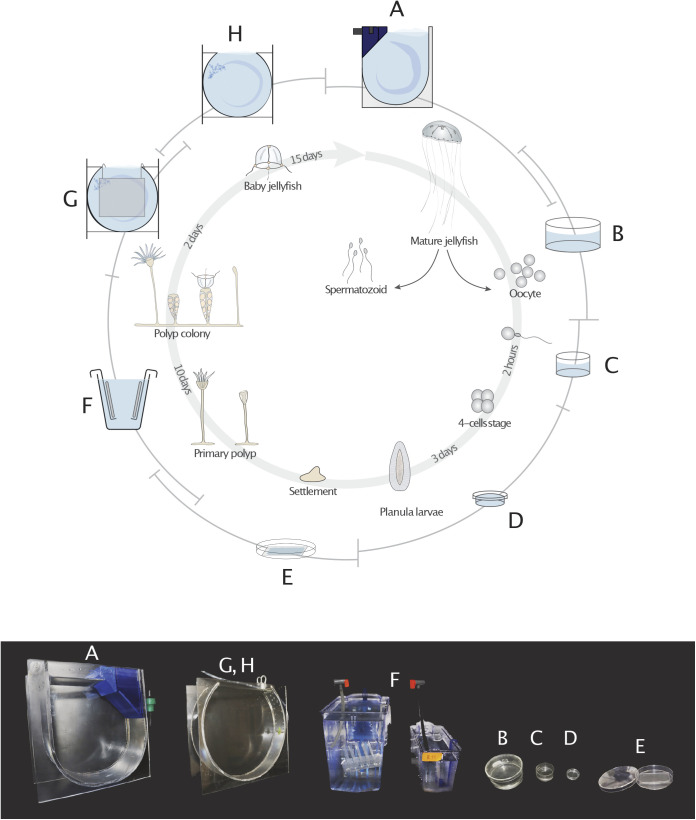
**Use of different culture tanks optimized for different stages of the *Clytia* life cycle.** (A) Mature jellyfish are maintained in Kreisel tanks (250×250×100 mm). (B) Male and female medusae can be transferred into crystallizing dishes (100 mm in diameter, 50 mm in depth) 1–2 h before spawning and maintained on a shaker (50–70 rpm). (C) Collected oocytes and sperm are transferred into smaller dishes (50 or 30 mm in diameter) to achieve fertilization. (D) 2–3 h later, developing embryos are transferred into small agarose-lined petri dishes. (E) Metamorphosis of planula larvae (60 h after fertilization or older) is induced on a glass slide (75×50 mm) placed in a plastic petri dish (100 mm in diameter). (F) Glass slides are transferred into polyp tanks once primary polyps have completed development (standard zebrafish tank 280×150×100 mm; 3.5 L or 280x 100x 60 mm;1.1 L) and maintained to grow polyp colonies. (G) Juvenile medusae budded from gonozooid polyps are collected by leaving glass slides in the nursery tank (280 mm diameter×90 mm) or in a crystallizing dish for up to 2 days. (H) The juvenile medusae are maintained in nursery tanks or in crystallizing dishes until their bell diameter reaches to 2.5 mm, large enough transfer to the Kreisel tank. See supplementary protocol for the detailed culture method.

**Fig. 3.**
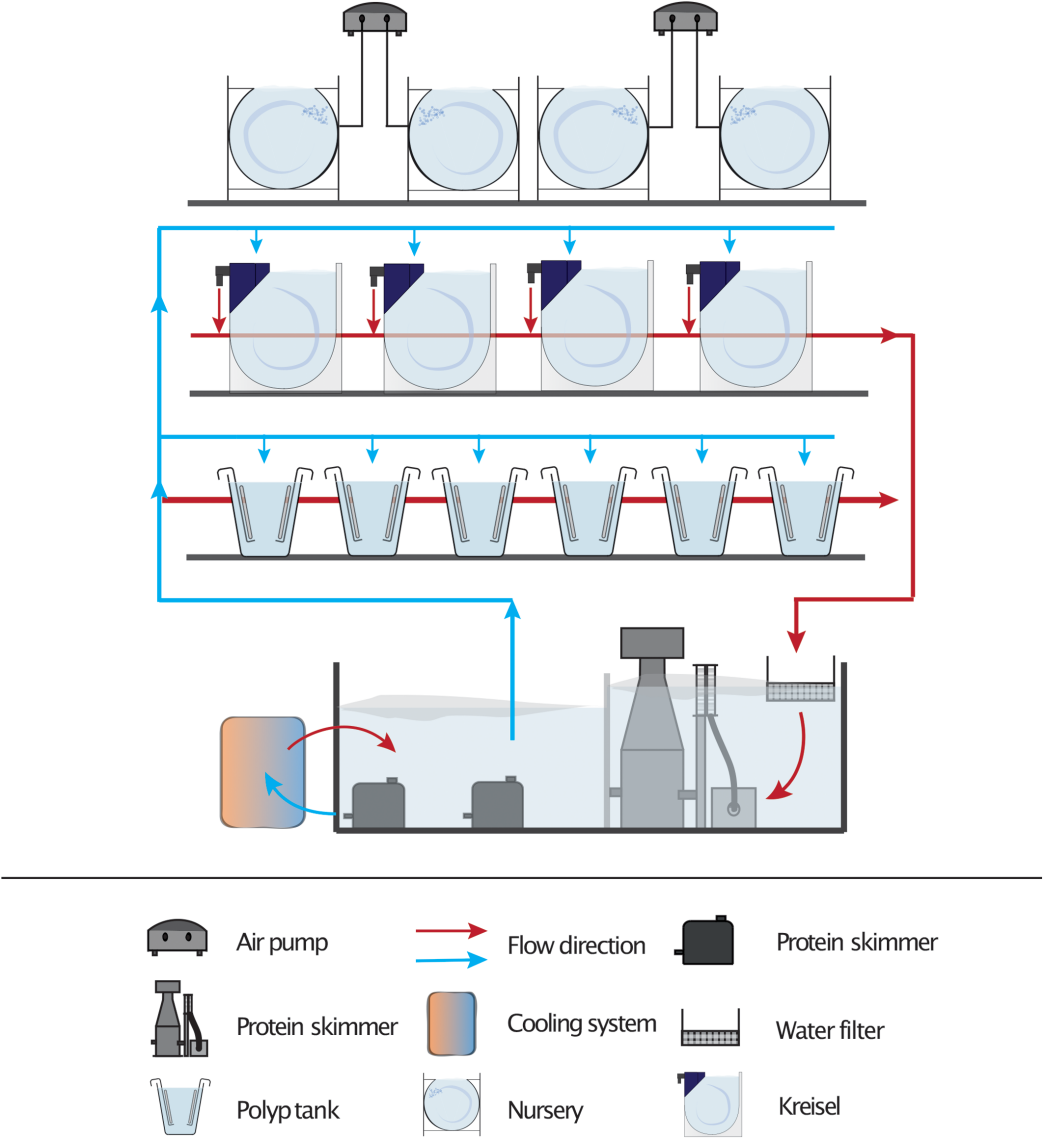
**Culture system in the laboratory.** Both Kreisel tanks (large medusae) and polyp tanks are integrated in a closed-circuit water aquaculture system. Water is supplied to each tank from the reservoir (50–80 liters) by a submerged pump. Excess artemia, food leftovers or released *Clytia* jellyfish are primarily removed by a nylon mesh (200 µm) or filter pad (50 µm), then by protein skimmer, before the water returns to the reservoir. The temperature is adjusted by water chiller/heater. The salinity is measured every 2–3 days and adjusted by adding deionized water. Roughly half of the water in the reservoir tank is replaced every 2 months.

Tank design was found to be critical for stable *Clytia* culture, and we optimized different culture tanks for each life-cycle stage (Fig. 2). Continuous water flow must be ensured to maintain the planktonic *Clytia* medusae (Fig. 1A) in the water column as they remain in the bottom and die without water flow. We originally used 5 L beakers with horizontal water rotation created by a paddle and a geared motor (5–6 rpm, Fig. S2F–H) similar to *Oikopleura* culture system (Bouquet et al., 2009), which was easy to set up but required intensive maintenance if used for large-scale *Clytia* culture. We thus devised a simple, closed-circuit aquarium system (Fig. 3) that accommodates major part of the life cycle, polyp colonies and medusae larger than 2.5 mm.

In order to generate embryos, medusae were temporary stored in 95 mm diameter crystallizing dishes (Fig. 2B) on a rotary shaker to collect eggs and sperm. For fertilization, collected gametes were mixed in smaller dish (3.5 cm diameter Fig. 2C) and embryos were cultured in a 2% agarose-coated plastic dish (1.5–3.5 cm diameter Fig. 2D) until reaching the planula larva stage (Fig. 1B). Planula larvae were induced to undergo metamorphosis (Fig. 1C) on glass slides (Fig. 2E) to form polyp colonies (Fig. 1D), which were maintained in polyp tanks (Fig. 2F). Juvenile medusae are formed by budding from these colonies (from gastrozooid polyps) and are constantly released into the sea water. They were collected by keeping colony plates in a drum shaped ‘nursery tank’ (Fig. 2G), in which water rotation was created by air bubbles, or in dishes (Fig. 2B). They were then grown in the same tank after removing polyp colonies (Fig. 2H) to 2.5 mm bell diameter size, at which point they were large enough to transfer into the ‘Kreisel tank’ (Fig. 2A), which is a key aquarium element of the jellyfish culture system (Greve, 1968; Raskoff et al., 2016). We reduced the cost of the Kreisel tanks by combining a simple U-bottom tank in poly(methyl methacrylate) (Fig. 2I) with a 3D-printed filter and nozzle parts (Fig. 2J). Details of these tanks and water-circulating system are explained in Materials and Methods.

### Growth of *Clytia* jellyfish and polyp colonies

We first characterized *Clytia* Z4B female medusa growth using the standard culture set up (see supplementary protocol). *Artemia salina* nauplii were used to feed twice a day (Fig. 5A). The newly released jellyfish took 6 days to grow to 2.5 mm bell diameter indicated by the dotted line 1, when all medusae were transferred to a Kreisel tank. After the transfer, the growth of the medusae accelerated, exhibiting a dual-phase growth curve (Fig. 5A). Density was reduced to 50 medusae per tank (roughly ten medusa per 1 L) once they grew to 5.0 mm bell diameter (dotted line 2). The medusae reached adult size (10–12 mm) 14 days after release, when ovulation was also observed.

**Fig. 4.**
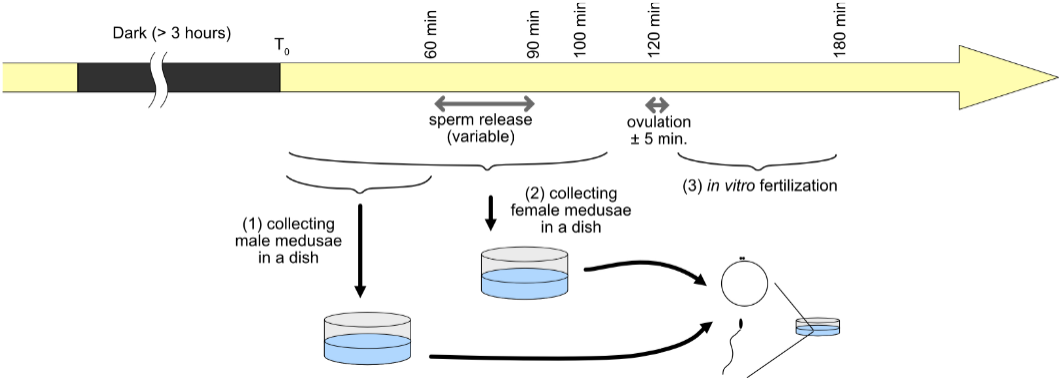
**Timing of egg and sperm collection and fertilization.** A daily schedule of jellyfish transfer, egg and sperm collection, injection and fertilization. See Materials and Methods for details.

**Fig. 5.**
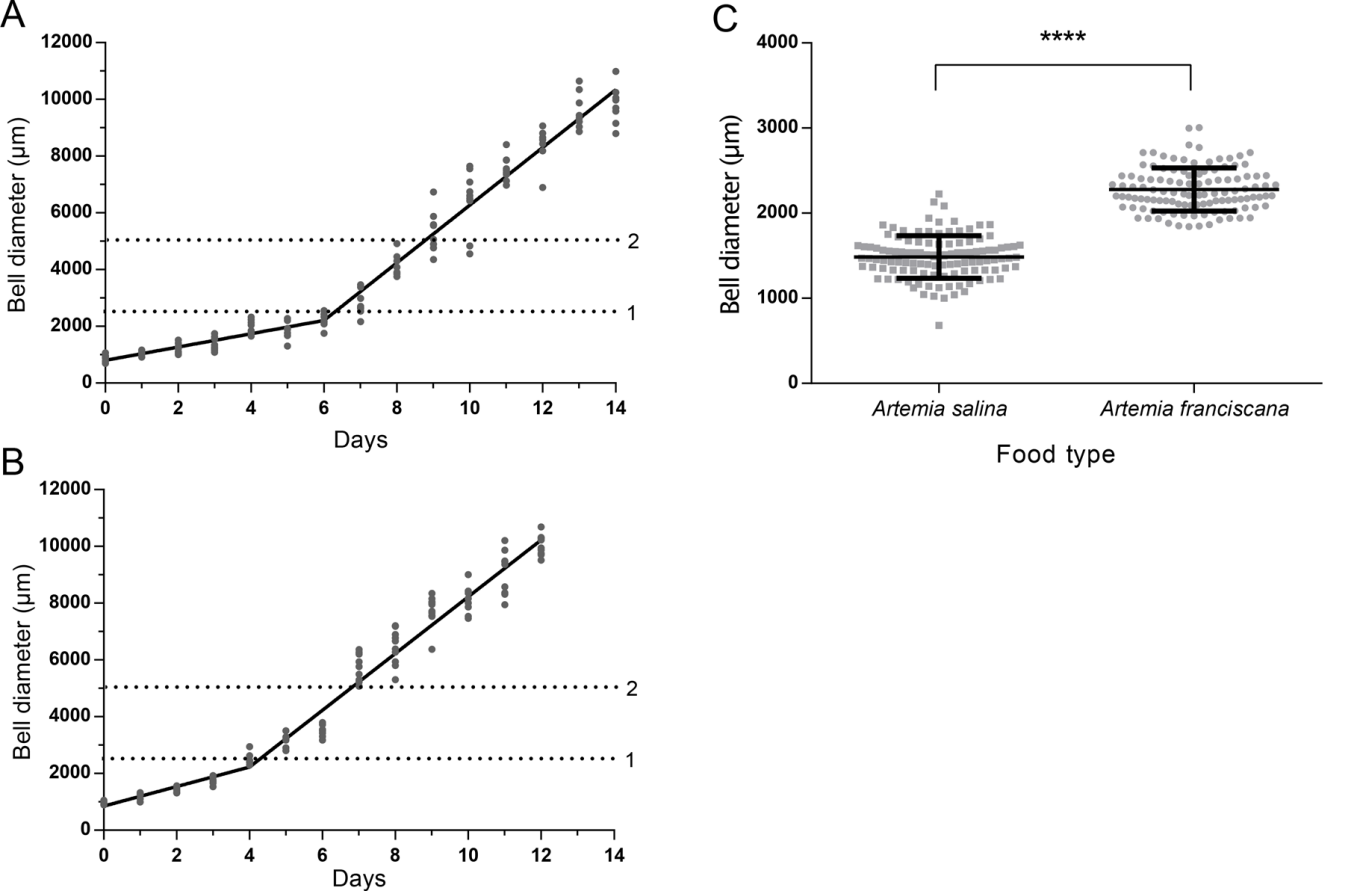
**Growth of *Clytia hemisphaerica* jellyfish.** (A) Growth of jellyfish fed with *Artemia salina* (segmental linear regression model, R²=0.98) and (B) with *Artema franciscana* (R²=0.98). Dotted lines 1 and 2 represent 2.5 mm and 5 mm bell diameters, the thresholds for transferring medusae from the nursery tank to the Kreisel tank and to decrease the density from more than 200/tank to 50/tank, respectively. Ten medusae were randomly chosen to measure the bell diameter each day. The segment boundary for two-phase growth was defined by date to transfer medusae. (C) Size comparison of 6-day-old jellyfish fed with *Artemia salina* and *Artemia franciscana* (*n*=115). Vertical bars correspond to standard deviation. Asterisk indicates significant difference of mean size (*t*-test ****P<0.0001).

To test whether the relatively slower growth observed over the first days spent in the nursery tank was due to the lower capacity for food capture in the juvenile stage, we assessed different food types. We found that smaller *Artemia franciscana* nauplii larvae, hatched from cysts produced in Vietnam, achieved the best growth of juvenile stage medusae (Fig. 5B). These *A.* *franciscana* nauplii are significantly smaller (body length 601±107 µm at third-instar larva stage) than standard *A. salina* nauplii (783±97 µm, Fig. S1). By feeding with *A. franciscana* nauplii, young jellyfish reached 2.5 mm diameter in 4 days instead of 6 days for *A. salina* (Fig. 5B). We directly compared the early phase of medusae growth between groups fed with these two types of Artemia (Fig. 5C). We cultured 115 baby medusa per tank and added equivalent volumes of *A. salina* and *A. franciscana* nauplii. The bell diameter of young medusa was significantly larger with *A. franciscana* 6 days later (Fig. 5C). These results suggest that feeding efficiency is critical for juvenile medusae growth. On the other hand, in later stage medusa the growth speed was similar between the two food types.

**Fig. 6.**
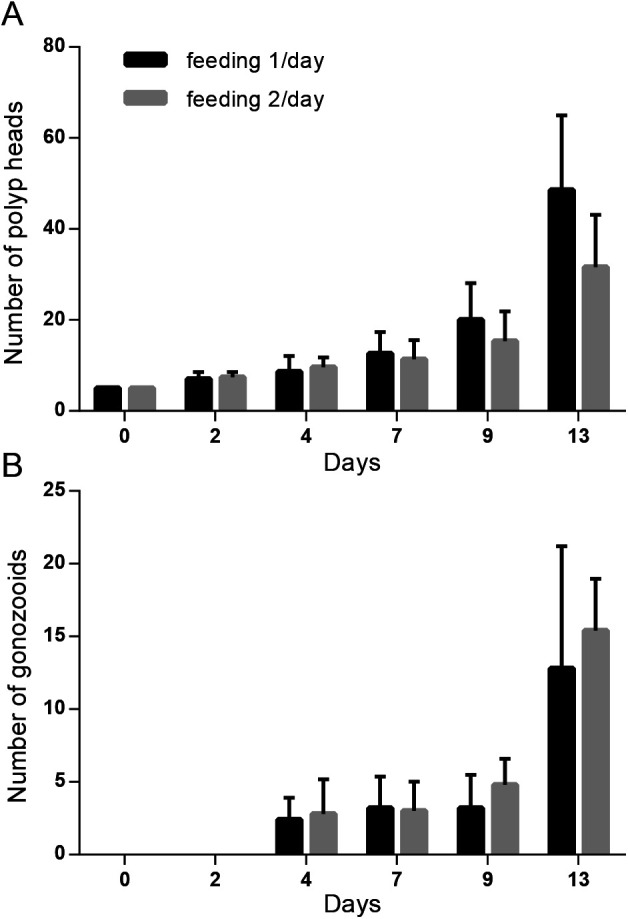
**Daily feeding is sufficient for growth of established *Clytia* polyp colonies.** (A) Polyp colony growth measured by number of polyps (gastrozooids) with different feeding frequency. 5 colonies (initially 5 feeding polyps/colony and has no gonozooid) are grown simultaneously for each feeding condition. (B) Number of gastrozooids created in the same colonies shown in A. Vertical bars represent standard deviation. No significant difference was detected by repeated-measures ANOVA with ad hoc detection power (1-β)=0.2.

Unlike medusa stages, the polyp colony grows asexually (vegetatively) and can be considered immortal, providing ideal material for conserving genetic strains. We measured the growth speed of the polyp colony under daily feeding by counting the number of gastrozooids (feeding polyps) and gonozooids (medusa budding polyps) (Fig. 6). We made transplant colonies of identical Z4B strains (see methods). Starting from colonies with five gastrozooids, the colony size roughly doubled to more than ten gastrozooids in 7 days and reached 30–40 gastrozooids in 13 days (Fig. 6A). The colonies formed a first gonozooid within 4 days and acquired more than ten gonozooids in 2 weeks (Fig. 6B). No clear difference in colony growth was observed between feeding once and twice per day. Feeding once per day is thus sufficient for the maintenance of wild-type colonies.

### Metamorphosis of planula larvae

**Fig. 7.**
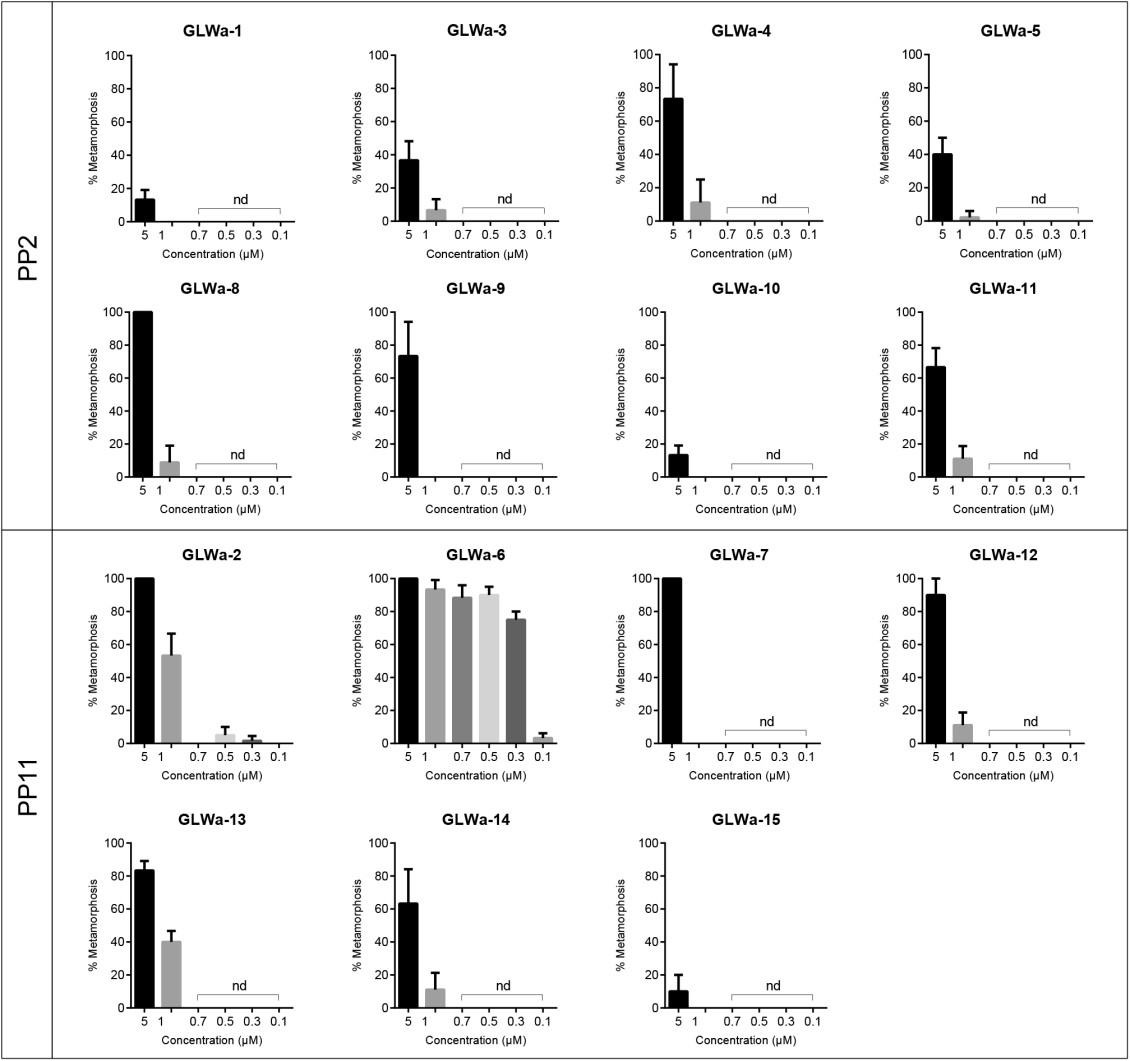
**Metamorphosis efficiency with different synthetic GLW-neuropeptides.** Synthetic GLW-amide neuropeptides predicted from the Che-PP11 and Che-PP2 precursors were synthesized and their metamorphosis inducing efficiency was tested. The list of peptide sequence is in Table 1. Experiment was repeated five times for each condition with 20 planulae for each repeat. Vertical bars represent standard deviation.

Once they have reached adult size with mature gonads, *Clytia* medusae can release eggs and sperm daily for several weeks following a dark-to-light cue, providing convenient material for studies of embryogenesis and larva formation, as well as for the creation of gene-edited *Clytia* lines. A critical step in generating mutant strains is to induce and successfully complete metamorphosis of planula larvae into primary polyps. In natural conditions, metamorphosis is triggered by unknown cues from bacterial biofilms, and involves downstream cellular responses mediated by GLW-amide family neuropeptides (Takahashi and Takeda, 2015). In the laboratory, a synthetic GLW-amide neuropeptide, GLW-amide-2 (GLWa-2, GNPPGLW-NH2), identified from the *Clytia* transcriptome sequences, had been used to trigger metamorphosis (Momose et al., 2018; Quiroga Artigas et al., 2018). The GLW-amide-2 sequence is similar to known hydrozoan metamorphosis-inducing peptides like metamorphosin A (EQPGLW-NH2) for *Hydractinia echinata* (Leitz and Lay, 1995) and KPPGLW-NH2 for *Phialidium gregarium *(*Clytia gregarium*) (Freeman, 2005), which, however, could induce metamorphosis, but at considerably lower efficiency (40–60%) than reported for *Clytia gregarium* (Freeman, 2005). We then sought to identify more efficient GLW-amides. Published *Clytia* neuropeptide precursor sequences (Takeda et al., 2018) include two pro-neuropeptide genes (*Che-pp2* and *Che-pp11*) that each encode several potential GLW-amide precursors. We tested 15 chemically synthesized neuropeptides (Table 1) derived from these sequences to see whether any were more active than GLW-amide-2. While most of the variants were able to induce metamorphosis at 5 µM, none were efficient at 1 µM or lower concentration, except for GLW-amide-6 (GLWa-6, pyroGlu-QQAPKGLW-NH3), which was active at as low as 0.3 µM, inducing virtually all planula to settle on the glass slide (Fig. 7) or other materials selected, depending on the following experiments (Table 3). GLW-amide-6 is thus, so far, the most effective inducer of *Clytia* larval settlement and metamorphosis *in vivo*.

**Table 1.**
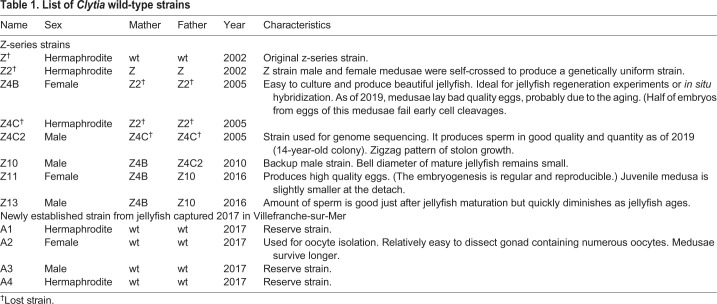
**List of *Clytia* wild-type strains**

**Table 2.**
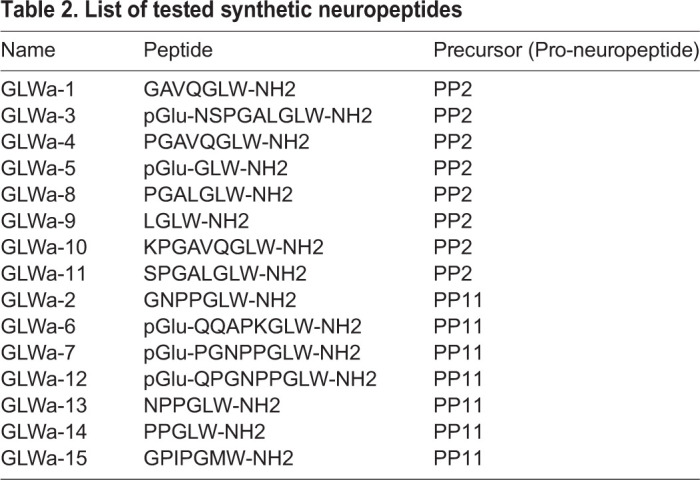
**List of tested synthetic neuropeptides**

**Table 3.**
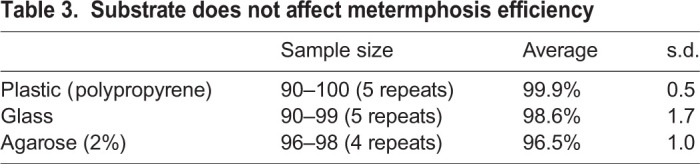
**Substrate does not affect metermphosis efficiency**

**Fig. 8.**
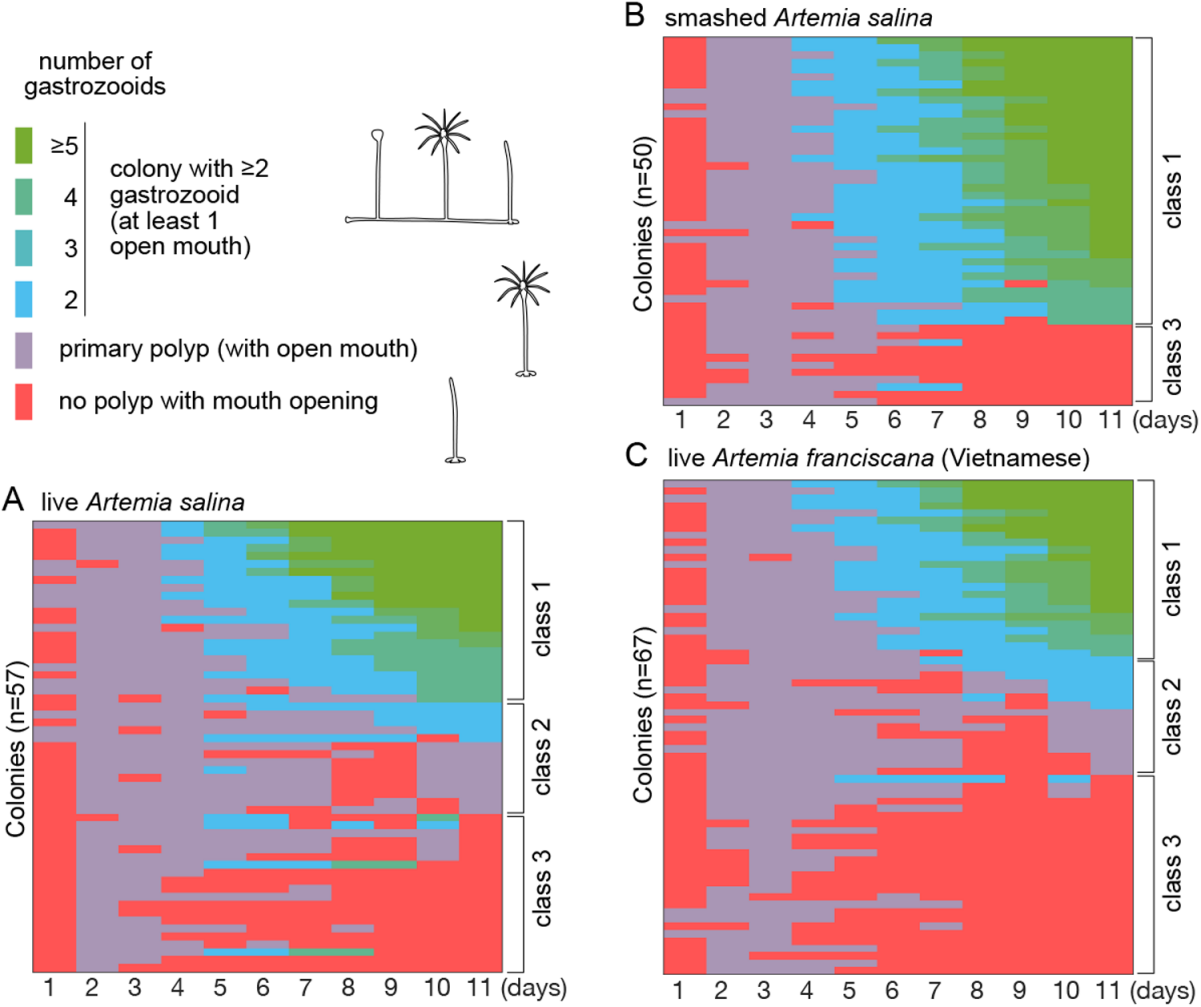
**Efficient colony formation is dependent on the early feeding.** The progress of colony formation successfully settled primary polyps under different feeding types was observed every day for 11 days. We fed (A) standard live *Artemia salina,* (B) smashed *A. salina* and (C) live *Artemia franciscana*, twice a day. The state of each colony was classified by the number of total polyps in the colony and the presence of at least one gastrozooid polyp with tentacles and mouth opening. Colonies are vertically aligned by the final polyp numbers in descending order. Each gastrozooid may repeat degradation and regeneration (see Supplementary data) in a single gonozooid. The patterns of polyp growth were classified into three groups, notably, class 1: straightforward colony formation including ‘stable’ polyp colonies (≥5 polyps) and promising colonies (three or four polyps), class 2: live colony futilely repeating gastrozooid renewal, which may eventually die, and class 3: lost polyp head after once making functional primary polyp and unable to regenerate (practically dead).

Survival and successful growth of the primary polyp into a multi-polyp colony has been a hurdle for making polyp colonies. Only about 10–20% of successfully settled primary polyps formed a polyp colony in a few weeks and the rate was highly variable in the same cohort. This has been a significant bottleneck for mutant colony production of transgenic animals and mutants created for instance by CRISPR/Cas9 technology. As the size of the primary polyp is usually smaller than that of gastrozooids within a colony (Fig. 1), we hypothesized that food capture may be again the key difficulty. We thus compared primary colonies fed either with intact *A. salina* larvae or with smashed ones using a 25-gauge syringe needle just before feeding. We also compared smaller *A. franciscana* larvae. The growth and survival of the primary polyp were observed for every 24 h for 11 days (Fig. 8). 60% of the primary polyps turned into polyp colonies with at least five gastrozooid polyps in 11 days (Fig. 8B, Table 4) significantly higher than those fed live non-smashed *Artemia* (25%, Fig. 8A,C). Functional polyp heads (gastrozooid heads with tentacles) were formed on day 2 or 3 in all conditions. Some of them arrested or died after losing polyp head (class three) or futilely repeated formation and degradation of the polyp heads (class two). With smashed artemia, the population of class three colonies was reduced, and no class two colonies were observed, suggesting the initial feeding of smashed food adapted to primary polyp is critical to boost subsequent polyp growth. The effect of food type was visible as early as 6 days after metamorphosis (Fig. 8) as a significantly higher rate of second polyp formation. Second polyp formation within 6 days was tightly associated with successful colony formation in later days (Table 5), suggesting that the primary polyp is dependent on food intake in the first few days, thus being necessary to extend a stolon and make a colony of multiple gastrozooids.

**Table 4.**
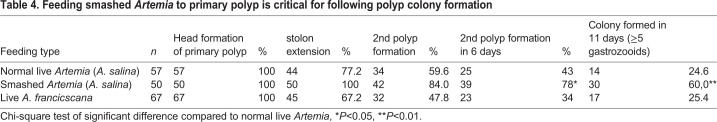
**Feeding smashed *Artemia* to primary polyp is critical for following polyp colony formation**

**Table 5.**
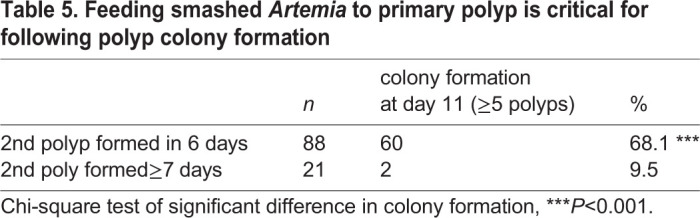
**Feeding smashed *Artemia* to primary polyp is critical for following polyp colony formation**

### Storage of polyp colonies

Vegetatively growing, regularly fed *Clytia* polyp colonies can be maintained for a long period of time without a genetic cross. Some hydrozoan species, such as *Cladonema pacificum,* can be maintained at 4°C for several months to years in a dormant state (personal communication from Ryusaku Deguchi). Developing such colony dormancy methods in *Clytia* would allow maintaining genetic strains for long periods with minimal care. We found that after one month of storage at 4°C, *Clytia* colonies lost polyps and did not recover when cultured at 18°C. In contrast, functional gastrozooids survived in all colonies stored at 10°C for one month, and were able to eat and grow immediately after transferring to 18°C, indicating that *Clytia* colonies are resistant against starvation when maintained at low temperatures (Fig. S3).

### Elimination

To avoid any risk of contamination of laboratory strains into the natural environment, it is important to eliminate any living material before discarding. We found that an effective method to euthanize *Clytia* planulae, polyps and medusae is exposure to low salinity water. Medusae and larvae disintegrated completely within 5 min when soaked in low osmolarity water (3.7‰ x1/10 salinity, Fig. S4A). The presence of the protective theca renders the polyp colony stage more resistant, but 1 h of incubation was enough to fully dissolve polyp colonies (Fig. S4B).

## DISCUSSION

We have developed a culture system for *Clytia hemisphaerica*, one of the hydrozoan model species suitable for developmental biology. The presence of a medusa stage in the *Clytia* life cycle opens many research possibilities. Its transparent body is suitable for cellular studies, including fluorescent microscopy. Medusae mature rapidly and spawn gametes once a day, reliably induced by a simple dark–light cue (Quiroga Artigas et al., 2018). In this work, we described an optimized *Clytia* whole life cycle culture system, developed so that biologists can reliably maintain and produce animal resources in the laboratory. The shortest full sexual generation cycle time for genetics studies in this system is about 2 months. A genetic strain can be maintained for many years as a polyp colony and can be shared by making colony duplicates.

### Medusa growth: the importance of early stage feeding

In our experience, a colony established on a large (75×50 mm) slide maintained in good nutritional conditions will typically produce several dozen baby medusae each day. The measurements reported here revealed that the medusae grows to adult size and sexual maturity (about 10 mm in diameter) in less than 2 weeks, a time course suitable for genetic studies. The first few days, i.e. until the juvenile medusae reaches 2.5 mm bell diameter, were found to be critical for successful jellyfish growth for two reasons. Firstly, these small medusae cannot be accommodated in the Kreisel tanks as they pass through the nylon retaining filters. Closed tanks such as bubble-circulating nursery tanks or crystallizing dishes can be used for this stage, though they require additional attention to the water quality with overfeeding being particularly harmful. Alternative systems for the early step of medusa culture could be developed in the future, for instance, to avoid the harmful effect of air bubbles that can damage medusae as they reach larger sizes. Secondly, juveniles cannot efficiently catch live *A. salina* nauplii. This issue was mitigated by using the smaller nauplii of *A. franciscana*, which significantly improved the growth of baby medusae and shorted the time to adulthood. Inefficient feeding may potentially be problematic for some mutant strains, for instance with developmental or neuronal phenotypes. Direct delivery of food to medusa mouth (hand feeding) may be required in this case.

### Maintaining the polyp colony

Another advantage of *Clytia* as a genetic model animal is the robustness of the polyp colony stage. A colony can rapidly extend stolons and generate new polyps in a vegetative manner. Commonly available *Artemia* nauplii are convenient and sufficient as food. Our tests showed that the colony size can double in less than 1 week, and that feeding once a day is sufficient, at least for wild-type strains. Colony growth does not seem to be limited by age or number of polyps. For example, the Z4B strain used in this work was established in 2005 and still propagates rapidly (Leclère et al., 2019). The polyp colony stage is thus useful for long-term maintenance of genetically modified strains created by CRISPR/Cas9 technology.

The standard aquaculture system described here uses artificial sea water (37‰) adjusted to the salinity of the Mediterranean water of the bay of Villefranche-sur-Mer, France, where our *Clytia* founder animals were collected (http://www.obs-vlfr.fr/data/view/radehydro/std/; Leclère et al., 2019). The species *Clytia hemisphaerica* is present worldwide and some local populations can certainly tolerate lower salinity, so colonies from animals collected from other localities might prefer other salinities. Medusae seems to be affected when the salinity reaches to 39‰ by evaporation. On the other hand, polyps from our laboratory *Clytia* strains were not largely affected when by accident the salinity increased up to 42‰ for a few days in our laboratory, although maintaining the salinity between 37‰ and 38‰ is highly recommended. Long-term physiological effects of extreme salinity remain to be examined. We maintain most colonies and jellyfish at about 18–20°C. Using systems maintained at 24°C during the early stages of polyp colony growth seems to favor irreversible determination to female. Medusa sex is partly determined by colony temperature according to the studies using hermaphroditic strains (Carré and Carré, 2000). Temperatures higher than 24°C seem to be harmful for polyp colony survival (unpublished observations). At lower temperatures, some hydrozoan species undergo dormancy, making dormant structures such as podocysts or dormant coenosarc (stolon) (Calder, 1990; Thein et al., 2012). *Clytia* colonies could survive for 1 month of starvation when they were kept at 10°C, retaining smaller but functional polyps, while regular feeding was necessary to maintain polyps at 18°C. Considering the lowest winter sea water temperature (12°C at 1 m and 50 m depth) where the founder *Clytia* jellyfish for these colonies were collected (http://www.obs-vlfr.fr/data/view/radehydro/std/), it is likely to be a hibernation state. Further tests will be necessary to develop reliable long-term methods for strain maintenance including cryoconservation.

The *Clytia* polyp colony can grow by constantly extending stolons and increasing the number of polyps with an appropriate food supply. Constant growth by new stolon extension is critical to maintain the colonies because individual gastrozooids are not immortal, unlike the whole colony. Colony growth is sensitive to various factors. For instance, stolon extension can be blocked by algae covering the glass surface. It is thus necessary to remove the old part of the colony and clean glass surface covered by algae by wood toothpick or plastic scraper once every few months. Reducing luminosity or temperature of the culture environment prevents the growth of red algae. Further, occasional flushing of culture plates by pipetting will also help to reduce accumulation of organic particles such as food leftovers. Making duplicates of genetic strains by polyp transplantation (see Materials and Methods) provides a convenient way to share genetic strains in the community. Finally, *Clytia* polyps, which are potentially invasive given their extremely high capacity for vegetative growth, can be easily eliminated within 1 h of adding 10 volumes of tap water, when a part of the colony is detached from the culture plates. Other physical or chemical methods such as UV-C irradiation or sodium hypochlorite treatment could also employed in transgenic *Clytia* facilities.

### From embryos to polyp colonies

Once they become sexually mature (2­–3 weeks after budding from the gonozooid depending on feeding), adult medusae daily spawn eggs or sperm, depending on the sex, for several weeks. After fertilization, embryos develop to form simple ciliated planula larvae with an elongated ‘oral–aboral’ body axis. By using the newly designed synthetic neuropeptide GLW-amide-6, we were able to increase the planula settlement efficiency to nearly 100%, which used to be variable and as low as 40% in the previous conditions (Fig. 7, 1 µM GLW-amide-2). Different settlement material can be used depending on the objective of metamorphosis. Another difficulty has been low survival rate after primary polyp formation to asexually growing polyp colonies. It is now clear that the initial feeding is critical for the second polyp formation and following colony growth. Feeding smashed artemia increased the colony formation efficiency from 25% to 60% (Fig. 8). This feeding improved the formation of secondary polyp (gonozooid) already at 6 days after the metamorphosis induction, indicating that efficient feeding in the first several days is critical to ensure the primary polyp survives and grows. The presence of unsuccessful primary polyps (class three, 25%) suggests there is a room for improvement, for example by choosing other food types. The key parameter seems to be food size, as the primary polyp is usually smaller than standard gastrozooid polyps in a colony. Smaller rotifers (less than 100 µm) were also tested. Primary polyps were, however, unable to recognize them as food (unpublished observation). Altogether, the colony formation efficiency was improved several times by the improvements documented here, which will facilitate gene KO by CRISPR/Cas9 and makes future genetic screens such as mutagenesis or gene trap feasible.

## MATERIALS AND METHODS

### Sea water

We used artificial seawater with salinity adjusted to 37‰ for all culture steps, which was prepared by dissolving 40 ***g*** (w/v) of RedSea salt mixture (https://www.redseafish.com/red-sea-salts/) in reverse-osmosis (RO) grade water (GE Merlin). For culture of embryos and planula larvae, sea water filtered by 0.22 µm Millipore filters (MFSW) was used. So far, the only commercial brand of sea salt that we have used successfully is RedSea Salt, which is reconstituted partially from natural sea salt. When we used chemically reconstituted artificial sea water, we observed ovulation failure of jellyfish. Salinity of sea meter was checked regularly using a digital reflectometer (PR-100SA, Atago). If salinity increased to 38‰ or higher due to evaporation, RO water was added to the culture system reservoir to adjust to 37‰. *Clytia* can survive for short periods (a few days) in a salinity range of 35‰ to 42‰.

### Temperature control and sex determination

Our standard culture temperature was 18–20°C. At 25°C, medusae grow poorly (Matsakis, 1993). Polyp colonies showed a similar temperature preference. At 24°C, colony growth was significantly slower. The mechanism of sex determination in *Clytia* is not known, but is influenced by the culture temperature of newly-established polyp colonies (Carré and Carré, 2000). We thus culture primary polyps either at a low temperature (18°C) that slightly favors establishment of male colonies (i.e. colonies releasing exclusively male jellyfish) or a high temperature (24°C) to favour the formation of female colonies.

### Water-circulating aquarium system

The closed-circuit aquarium system accommodated polyp tanks and Kreisel tanks (Fig. 2A,F and Fig. 3) build on the salt-resistant aluminum shelving (Fermostock, Fermod). The water temperature was adjusted by a water cooling/heating system (TECO TK-500) installed in the reservoir. The walls and floors of the shelving were covered by black PVC plates (5 mm). The waterproof LED lighting (LED ribbon with SMD 5050 chips) was installed on the wall behind the tank to allow the transparent *Clytia* jellyfish to be visible with pseudo-dark-field illumination. In order to limit growth of red algae in the culture system, polyp colonies were maintained in the dark or under reduced ambient light. Sea water was distributed to each tank by a submersible pump (ex Eheim universal 3400) through PVC pipes. All overflown sea water was recycled to the reservoir, after eliminating particles by passing it through nylon mesh (opening size 200 µm) or a filter pad (Combo filter pad classic bonded and 50 micron fine water polishing filter, Aquatic Experts). In addition, a protein skimmer (H&S aquaristik, A150-F2001) and UV-C lamp (Eheim Reeflex 9 W) were installed in the reservoir to eliminate smaller particles and parasitic organisms, respectively. Plastic substrates (polyether sponge, 50 mm thickness and 0.5 mm pore size) were placed in the reservoir to grow and maintain ammonia- and nitrite-fixing bacteria.

### Polyp tanks

We used commercial zebrafish tanks (Fig. 2F, 1.1 L model ZB11CP or 3.5 L model ZB30CP, Tecniplast) for polyp stages. Polyp colonies growing on glass slides (standard: 75 mm×25 mm or wide: 75 mm×50 mm) or larger glass plates (80 mm×60 mm or 160 mm×140 mm) were suspended on the side of the tank in histology slide baskets (Kartell, 20-Slide Microscope Slide Staining Dish, 235405) or 3D-printed plate holders (https://github.com/momotsuyo/Clytia-aquarium). Sea water was supplied continuously to each tank at a rate of 0.5–1.0 L/min. Prior to the first use, these polycarbonate plastic tanks were thoroughly conditioned by soaking in sea water for 3 months to remove residual chemical contamination.

### Kreisel tanks for jellyfish

We cultured adult medusae (over 2.5 mm size, typically achieved in 6 days after release) in a modified version of the Kreisel tank (Fig. 2A) described by Greve (Greve, 1968; Purcell et al., 2013), simplified and optimized to maintain *Clytia* medusae healthily. The main tank was built from Polymethyl methacrylate (PMMA) plates (Fig. 2I). Water nozzle and filter parts were made by 3D printing using polyethylene filament (Fig. 2J, Volumic3D, PET-G Ultra filament, https://www.imprimante-3d-volumic.com/). The 3D models are available at https://github.com/momotsuyo/Clytia-aquarium. These two parts were assembled using low-cost garden watering pipe connectors (L-form connector for 16 mm watering pipes with ¾ inch thread, see Fig. 2B). The speed of water rotation was adjusted to 10–20 mm/second near the tank periphery, obtained by regulating the flow rate to 150–300 ml/min, depending on the opening diameter of the nozzle (1.5–2 mm×4 nozzles, shown in red arrows in Fig. 2J) to create water flow parallel to the mesh plane.

Typically, *Clytia* medusae 2.5 mm diameter (about 5–7 days after budding) or larger were hosted in our Kreisel tanks and could be maintained up to 5–6 weeks until their natural death. Up to 200 small (2.5–5 mm bell diameter) jellyfish may be maintained in one tank equipped with a fine mesh filter (200 µm mesh size). Once they had grown to 5 mm bell diameter or more, they were redistributed into multiple Kreisel tanks with coarse mesh filters (1 mm mesh size) at 40–50 jellyfish/tank.

### Nursery tanks and crystallization dishes for juvenile jellyfish

For some culture needs, notably for young medusae and embryos, we used individual tanks and dishes. A drum-shaped ‘nursery’ tank was used to collect (Fig. 2G) and grow (Fig. 2H) juvenile medusae up to 2.5 mm diameter. Medusae larger than this size grew better in Kreisel tanks (see above). Vertical water circulation was created by air bubbles (100–200 ml air/minutes) from 5 mm tubing inserted at one side of the shell, supplied by an air pump. Juvenile medusae were alternatively collected and raised in 95 mm crystallization dishes (Fig. 2B), this method being preferable if the number of medusae was low (less than 100). These dishes were kept on a rotary shaker set at 50–70 rpm (Fig.S1E). The sea water in the dish was changed within a few hours after feeding. The dishes were also used to temporarily concentrate adult jellyfish during spawning for short periods (up to a few hours) to allow gamete collection.

### Aquarium maintenance

Feeding once or twice a day respectively ensures effective polyp and jellyfish growth colonies can support a few days of starvation. Salinity adjustment and filter cleaning was performed every few days. Kreisel tanks were cleaned every 1–2 weeks, depending on debris accumulation, washing tanks briefly with tap water. Up to a half of sea water in the system was changed every few months. On occasions where complete cleaning of the aquarium system was performed, part of the used sea water was stored and reintroduced to maintain the aquarium microbiota. It was important to clean colony slides and plates once every 3–8 weeks to stimulate new colony growth and to remove old parts of the colony. This task needed to be coordinated with jellyfish collection as jellyfish production is reduced after colony cleaning.

### Feeding rational

For both polyp and medusa stages, *Artemia salina* (Sep-Art, Ocean Nutrition or INVE Aquaculture) nauplii, at least 1 day after hatching (third instar) (Copf et al., 2003), were used for feeding (Fig. S1JKN). Younger *Artemia* nauplii (Fig.S1ILM) contain indigestible yolk lipid and block stolon trafficking and eventually kills polyp colonies (T.M. unpublished observation). Smaller *Artemia franciscana* (Vinh Chau pond strain, Vietnam, also available as *Artemia* AF Sep-Art from INVE aquaculture Fig. S1O) (Van et al., 2014) were used for juvenile medusae less than 2.5 mm. Unless otherwise specified, we fed twice a day with a minimum 6 h interval. This study revealed that feeding once a day is enough to support growth of wild-type polyp colonies. We also showed that primary polyps and very young colonies (less than five polyps per colony) grow best when they were fed with smashed *Artemia*. It was essential to wash *Artemia* extensively with tap water before introducing them into the culture system as described below. *Artemia* cultures were a common source of bacterial or parasite infections, and *Artemia* hatched from shell-free cysts by chlorine treatment appear to reduce early death of medusae (B.W., unpublished observation).

### Preparation of *Artemia*

(1) Set up *Artemia* hatching cone (Fig. S1G) install a light source, an aeration and an aquarium heater with thermostat adjusted to a range between 25°C and 30°C. (2) Introduce 10 ml of *Artemia* cysts per 1 L and incubate for at least 24 h. (3) Stop the aeration and heater, wait for 5–15 min and collect hatched *Artemia* nauplii from the bottom of the cone. (4) In the case of Sep-Art *Artemia*, remove iron-coated cysts using a magnet (Sep-Art Separator, Ocean Nutrition). (5) Rinse *Artemia* nauplii with tap water then clean sea water using an *Artemia* sieve (120–180 µm mesh size) and maintain them in sea water with mild aeration at 18–22°C.

### Feeding of polyp colonies and medusae

(1) Prior each use, wash *Artemia* nauplii in the *Artemia* sieve under running tap water (5–10 s) and then resuspend in sea water. (2) Add *Artemia* to the jellyfish/polyp tanks using a pipette (Fig. S1H).

Slightly excess feeding was acceptable for tanks with circulating sea water (polyp tank and Kreisel tank with standard mesh), from where *Artemia* nauplii flow out. Otherwise, the amount of feeing needed to be carefully adjusted. In case of juvenile medusae in crystalizing dishes, sea water was changed a few hours later.

### Smashing *Artemia*

Freshly smashed *Artemia* nauplii are used to feed primary polyps and small colonies.

(1) After rinsing (see above), take concentrated *Artemia* nauplii (< 1 ml) in an Eppendorf tube and smash by passing them through a 25 G needle attached to a 1 ml syringe back and forth three to five times. This makes a viscous suspension. (2) Transfer the suspension to a small dish (3.5 cm diameter), add 5–10 ml sea water and wait for 3 min. (3) Take fragmented nauplii from the bottom with a Pasteur pipette and gently apply to primary polyps from 1–2 cm distance.

### Strain duplication by polyp cuttings

*Clytia* strains can be propagated via ‘cuttings’ of the polyp colonies onto fresh slides. The attachment of the cutting depends on new stolon growth and the efficiency is variable and usually low (typically between 10% and 50%).

(1) Feed the donor polyp colony several hours prior cutting to help new stolon growth. (2) Cut polyp (gastrozooids) from the colony at the bottom of the vertical stem (close to the stolon) with microscissors (ex. Moria surgical 8100) and collect them in a 3 cm dish. (3) Clean the polyp cutting by vigorous pipetting using a plastic transfer pipette. (4) Label new glass slides with a diamond pen and clean them with warm water (40–50°C). (5) Place slides in a 100 mm diameter petri dish with 30–40 ml sea water. Transfer 5–10 gastrozooids on the slides and leave them undisturbed for one night. (6) Carefully transfer the glass slides into a polyp tank. Feed immediately to support stolon extension, otherwise the transplanted gastrozooid may degrade, extending a stolon but failing to grow further.

### Colony cleaning

Cleaning of polyp colonies was important to maintaining genetic strains, since individual gastrozooids are not immortal but repeatedly formed and degrade. Allowing constant stolon growth and polyp formation is a safe way to maintain colonies in a good state. Regularly cleaning the surface of the glass slides and removing the old part of polyp colonies every 3–8 weeks creates clean, open space for new stolon growth. Cleaning slides also prevents colonies overgrowing and potentially contaminating other tanks. The strategy of cleaning depends on the speed of colony growth and cleaning frequency (Fig. S2A–E). There are two types of cleaning: conservative cleaning to maintain the maximum number of healthy polyps ideal for small or slow-growing colonies, and quicker, global cleaning (less than 5 min/slide) to eliminate the oldest (and contaminated) parts of colonies to make a clean space for new colony growth.

### *In vitro* fertilization

Kreisel tanks were maintained under a 24 h day–night cycle with a dark period of 3–8 h. Egg release from the gonads reproducibly occurs 110–120 min after the light illumination. Sperm release was usually earlier (around 60–90 min after light) and the timing was variable. In the protocol below, T_0_ represents the time of dark to light transition (Fig. 4).

(1) At T_0_+60 min or earlier, transfer male medusae to 95 mm-diameter dishes containing 150–200 ml of seawater (5–20 medusae/dish) and keep aeration on a rotary shaker (60–70 rpm). (2) At T_0_+100 min or earlier, similarly transfer female medusae to dishes. (3) Once ovulation is complete (at T_0_+120), transfer medusae back to the Kreisel tank, leaving eggs and sperm in the dish. (4) Eggs can be concentrated in the center of the dish by leaving the dish for 5–10 min on the shaker, with 60–70 rpm rotation. (5) Eggs are collected in smaller (3.5 cm) dishes for experiments or microinjection. (6) *In vitro* fertilization must be induced by 1 h after ovulation (T_0_+180 min). Take sea water from the bottom of the male dish and add it to dishes containing eggs. Sperm density should be adjusted following observation under dark-field illumination so that sperm can be visible around the eggs but not present in dense clouds. (7) After fertilization, developing embryos can be selected at the two- to eight-cell stage (1–2 h after gamete mixing) or the day after (gastrula stage) and transferred to MFSW and incubated at 20°C. MFSW supplemented with penicillin-streptomycin cocktail can be used to prevent bacterial growth that naturally induces uncontrolled metamorphosis (5 unit/ml penicillin and 5 µg/ml streptomycin, x1/2000 dilution of Sigma P0781).

Note that fertilization rates will be poor and embryonic development will be irregular if eggs are fertilized 2 h or more after ovulation. Sperm density should be adjusted if many embryos show cleavage failure due to polyspermy.

### Inducing metamorphosis

(1) Culture planula larvae in MFSW containing penicillin and streptomycin. (2) Wash the surface of large glass slides (75 mm×50 mm) with warm tap water (40–50°C) and dry. Place them in 100 mm diameter petri dishes. (3) Dilute GLW-amide peptide stock solution (1–5 mM in distilled water, stored at −20°C) just prior to use in MFSW (4 ml/large slide). Recommended final concentration for GLW-amide-6 is 0.5 µM. (4) Using a plastic transfer pipette, carefully pour 0.1 ml/cm^2^ or more peptide-containing sea water over the glass slide (4 ml for large glass slides, Fig. S1F). (5) Transfer 2.5–3-day-old planulae using a glass pipette. Disperse them as the settlement starts quickly. (6) Cover the dish with a lid to minimize the water evaporation and keep the dish undisturbed. (7) Planula will be firmly settled in several hours after peptide treatment. Transfer slides to polyp tanks, not later than 16 h after induction. (8) Start to feed smashed *Artemia* once the primary polyps have extended tentacles and open mouths. (9) Once the colony contains multiple gastrozooids, it can be fed normally with live *Artemia* nauplii.

### Animal strains

Currently available wild-type *C. hemisphaerica* strains are listed in Table 1. The main Z-series strains, originated from a single hermaphroditic Z strain, have been used so far in the *Clytia* user communities, including Z4B (female) and Z4C2 (male). The Z4C2 strain (male) was used for whole genome sequencing (Leclère et al., 2019).

### Measurement of medusa growth

Juvenile Z4B strain medusae newly released from the colony were collected in the nursery tank (up to 1000 medusae may be obtained in a 4 L tank after one night, depending on the strain and colony size). After removing the polyp colony glass plate, juvenile medusae were kept in the tank until they reached about 2.5 mm in bell diameter, before being transferred to a Kreisel tank. The density of medusae was reduced to 50–60 when they reached 5 mm in diameter. The bell diameter of ten randomly sampled jellyfish was measured using an Axiozoom microscope using Zen software (Zeiss). For direct comparison of the effect of food types on early medusa growth, the same number of newly released medusae (115 medusae per tank) were placed in two nursery tanks and cultured for 6 days, feeding twice a day with about 350 *Artemia* per tank (corresponding to three *Artemia* per medusa). The bell size of all medusae in the tank was measured on the sixth day.

### Measurement of colony growth

Transplants of the Z4B strain colony grown on standard glass slides were cleaned so that only a single stolon-connected colony with five gastrozooids remained on each slide. The colonies were fed once or twice a day. The colony size was quantified every second day for 2 weeks, as were the numbers of functional feeding gastrozooids and gonozooids.

### Efficiency of metamorphosis-inducing neuropeptides

Metamorphosis of planula larvae was induced by transferring them onto glass slides covered with at least 0.1 ml/cm^2^ of sea water containing synthetic neuropeptides (Fig. 2E). A selection of synthetic amidated peptides derived from the neuropeptide precursors Che-PP2 and Che-PP11 (Takeda et al., 2018) were tested as metamorphosis inducers (Table 2) on 3-day-old planula larvae obtained by crossing Z13 (male) and Z11 (female) strains. Planula attach firmly onto the slide within a few hours (Fig. 1C, left). Metamorphosis efficiency was measured by successful settlement on the slide at 12 h, as the timing of stalk extension is variable, from several hours to days.

### Growth of post-metamorphic primary polyps into polyp colonies

To measure the survival and colony-formation rate of primary polyps, the development of successfully settled planulae was monitored. For each primary polyps/colonies the numbers and states of gastrozooids and gonozooids were recorded every 24 h. See supplementary data for the stage of polyp formation/regeneration and their classification.

### Testing survival of polyp colonies at low temperatures

To test the ability of colonies to survive at low temperatures without feeding, we put Z4B colony in a 50 ml polypropylene Falcon tube containing clean MFSW and kept at 4, 10 or 18°C for 4 weeks without feeding. The sea water was changed once after 2 weeks. Viability of the colonies was scored by the presence of polyps with tentacles and confirmed by recovery of the polyp colony after 15 days at 18°C with regular feeding.

### Testing of treatments to efficiently euthanize *Clytia* polyps and jellyfish

To avoid environmental release of genetically modified *Clytia* strains, we examined effective ‘sterilization’ by osmotic shock. Polyps, medusae and planula larvae were incubated in 1/10 diluted artificial seawater by adding 10 volumes of tap water to the culture container and incubated for different times (1–5 min for medusae and planula; 30–120 min for polyps). Complete loss of living tissue was evaluated under the microscope immediately and 24 h after the treatment.

### Statistical analysis

Statistical analysis was performed using GraphPad Prism (www.graphpad.com) for jellyfish growth (Fig. 5) and R (r-project.org).

## Supplementary Material

Supplementary information
