# Supplementary material for: An improved whole life cycle culture protocol for the hydrozoan genetic model Clytia hemisphaerica
Source: Biol Open. 2020 Nov 5;9(11):bio051268. doi: 10.1242/bio.051268 (PMC7657476; doi:10.1242/bio.051268)
Supplement: Supplementary information [file biolopen-9-051268-s1.pdf]

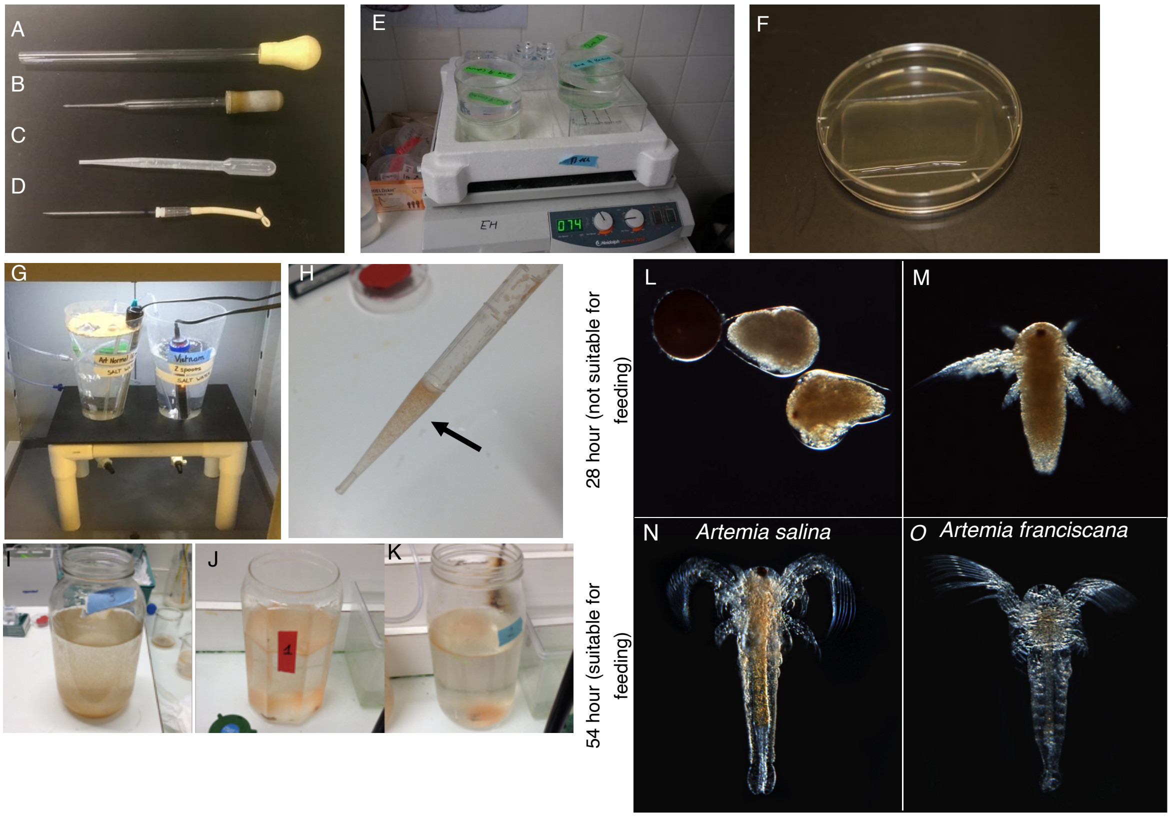

Figure S1. Additional information for Clytia culture: culture material and feeding
See Materials and Methods for detailed explanation. (A) Jellyfish transfer pipette: glass or plastic pipes (diameter 10 mm) with pipette bulb. (B) Pasteur pipette: for feeding and transferring embryos. (C) Disposable transfer pipette (3~5 ml size for cell culture, non-sterile, ex. Samco Scientific 3 ml "225": for feeding, fertilization (sperm transfer) or small medusae less than 5 mm. (D) Embryo/egg transfer pipette; made by pulling glass micropipette (ex Hirschmann 100 μl, cat no. 9600199) under alcohol lamp flame. The tip is cut with a diamond pen and flame rounded. Used with a pipette bulb (ex. Hirschmann cat no 9650101). (E) Short time (less than a few hours) culture of adult jellyfish on a shaker, for egg/sperm collection. This is also used to culture relatively small numbers (< 100) of juvenile jellyfish for several days (up to 2.5 mm). (F) Metamorphosis of planula on a glass slides.(G) Artemia hatchery setup (2 L Brine Shrimp Hatcher, ZM Fish Food & Equipment). (H) A typical amount of Artemia for feeding to a Kreisel tank with 50 adult jellyfish. (I-K) identifying Artemia stage for feeding by color and behavior of Artemia. (I) Artemia just after hatching and not suitable for feeding (28 hours of incubation, corresponds to L and M). Mostly sedimented on the bottom without aeration and indicate yellowish grey color. (J) 1day after hatching (corresponds to N), indicating vivid orange color. They are able to swim and some come to the surface by phototaxis.(K) 2 days after hatching. Actively swimming and show phototaxis. Both J and K are suitable for feeding. (L and M): Artemia salina after 28 hours of hatching. (N). *Artemia salina* nauplii1 day after hatching. (O) *Artemia franciscana* nauplii1 1 day after hatching.

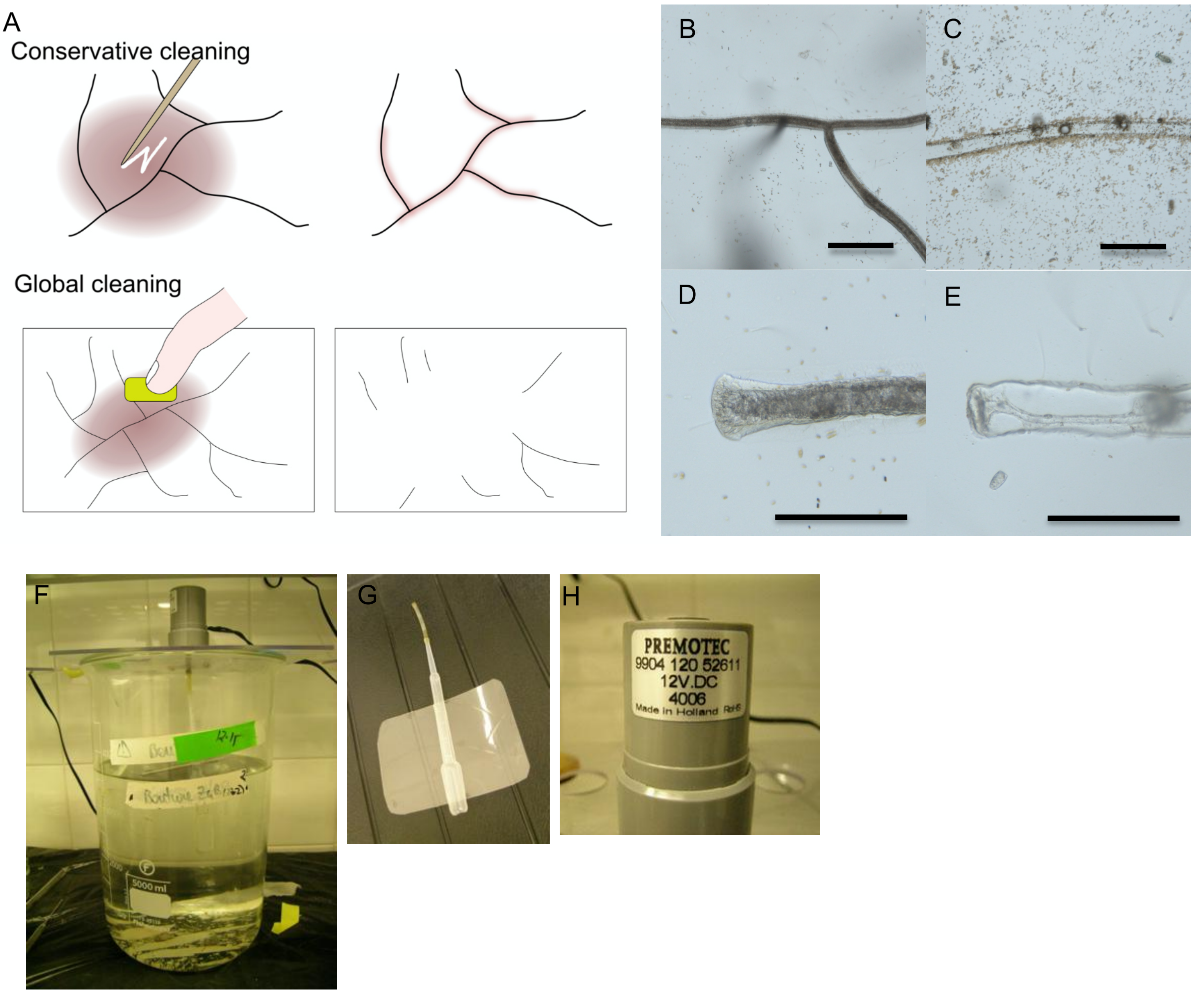

Figure S2. Additional information for Clytia culture: colony cleaning and small scale culture system
(A) colony cleaning strategies. If the colony is small or the strain grows slowly, conservative cleaning with toothpick is recommended, to remove algae or dirt keeping polyps. For most strains removing heavily contaminated part with a small piece of sponge is highly efficient. (B) Stolon in healthy state and without algae contamination, to be maintained. (C) Empty hydrotheca without stolon cells inside, to be eliminated by global cleaning, together with red algae growing nearby. (D) Actively growing stolon tip, which is characterized by “growth cone” morphology. (E) An example of growth-arrested stolon tip. This occurs by a various reasons including insufficient feeding or stolon clogging. In latter case, removing such part may stimulate stolon growth by reforming a growth cone. (F) A setup of 5-lier beaker c ulture. Water i shorizontally rotated by a fin (G) with 12 volt 5 rpm geared electric motor (H). The beaker can accommodate both polyps and jellyfish, and are thus suitable for small scale tests. This approach however is more labor-intensive for large scale cultures as the sea water should be replaced manually once or twice a week, and the food concentration must be closely controlled to prevent accumulation of dead Artemia. Bars= 500 µm

Biology Open • Supplementary information

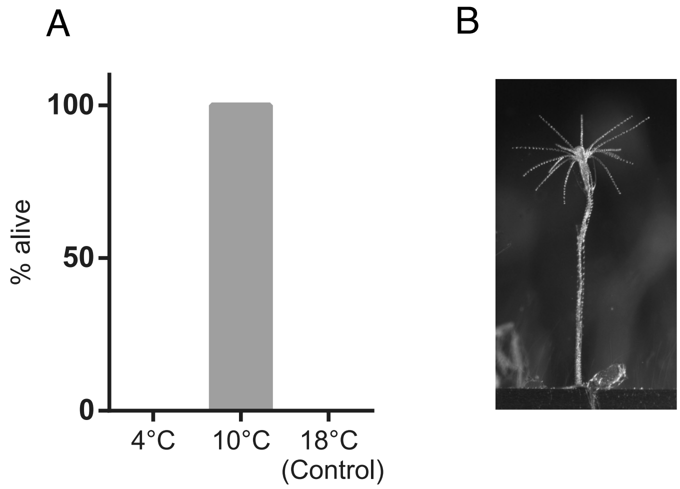

Figure S3. "Hibernation" storage of polyp colonies under starvation at lower temperature. (A) Proportion of living polyps colonies (N=5 each) stored for one month at 4°C, 10°C and18°C without feeding. (B) An example of a polyp after one month at 10°C. Potentially allowing short time storage of genetic strains as polyp colonies.

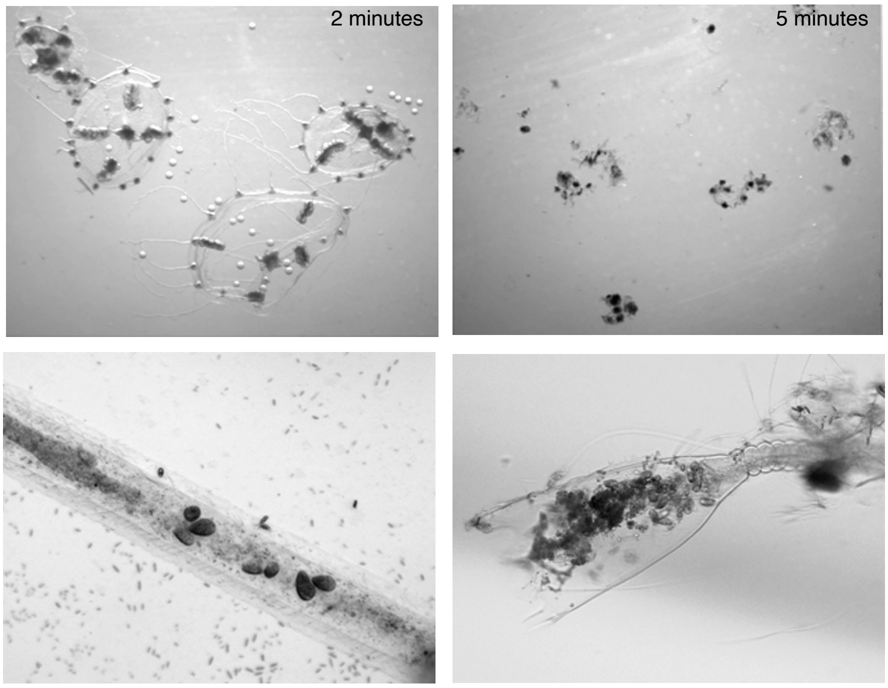

Figure S4. Sterilization of *Clytia* jellyfish and polyps prior disposal.
Mature jellyfish incubated for (A) 2 minutes with tap water (ten times diluted sea water) and (B) 5 minutes, showing minimum 5 minutes incubation in fresh water is sufficient to fully dissolve jellyfish. Longer incubation (1 hour) is required to sterilize (C) stolon and (D) gastrozooid due to chitinous theca protecting them.

Data S1

Click here to Download Data S1
